# Prospective comparative validation of a novel endoscopy chair and endoscope manipulation apparatus (EasyFlex) for ergonomic improvement in fURS procedures

**DOI:** 10.1186/s12894-026-02200-7

**Published:** 2026-05-29

**Authors:** Serdar Toksoz, Alper Asik, Yalcin Kizilkan, Samet Senel, Burak Koseoglu, Erhan Erdogan, Kemal Sarica

**Affiliations:** 1 Urology Department, Sincan Research and Training Hospital, Ankara, 06180 Türkiye; 2 Urology Department, Sancaktepe Research and Training Hospital, Istanbul, Türkiye; 3 Urology Department, Ankara Bilkent City Hospital, Ankara, Türkiye; 4 Urology Department, Polatli Duatepe State Hospital, Ankara, Türkiye

**Keywords:** Endoscopy Chair, Endoscopy holder apparatus, EasyFlex, Ergonomic surgery

## Abstract

**Background:**

This study aimed to validate the functionality and usability of a doctor’s endoscopy chair and an endoscope carrying/manipulation apparatus known as the EasyFlex.

**Methods:**

Prototype models of the EasyFlex apparatus were evaluated by five expert urologists specializing in endourology at three medical centers. The surgical and ergonomic advantages of the doctor’s endoscopy chair and the endoscope carrying/manipulation apparatus were compared with those used during traditional flexible ureterorenoscopy (fURS) procedures for kidney stones, with ergonomic outcomes assessed using the Ergomini scale.

**Results:**

The ergonomic evaluation of both systems revealed that surgeons experienced significantly greater ergonomic discomfort during traditional fURS procedures compared to those using the EasyFlex system, as indicated by higher total ergonomic scores (37.6 vs. 12.6, *p* < 0.001). Furthermore, the EasyFlex group demonstrated a significantly lower rate of intraoperative complications than the traditional fURS group (11.8% vs. 27%, *p* = 0.017).

**Conclusion:**

Compared with the traditional approach, the results obtained in our study demonstrated that the EasyFlex apparatus could provide superior ergonomic conditions during fURS procedures. Additionally, the rate of intraoperative complications was lower in the EasyFlex group.

**Supplementary Information:**

The online version contains supplementary material available at 10.1186/s12894-026-02200-7.

## Background

Recent advances in laser technology and flexible ureterorenoscopy (fURS) have resulted in remarkable improvements in kidney stone surgery [1]. Retrograde intrarenal surgery (RIRS) has increasingly become an alternative to traditional stone management approaches such as extracorporeal shock wave therapy (SWL) and percutaneous nephrolithotomy (PNL), particularly in the management of medium-sized (10–20 mm) renal calculi [2]. Improved imaging quality coupled with the flexibility of the scope used has increased the popularity of procedures in which an increasing number of urologists are involved in kidney stone management [3, 4]. Notably, as a procedure with a lower learning curve for endourologists, fURS provides a minimally invasive, efficient, and reliable approach. Despite such advantages, the fURS procedure has been found to be demanding, particularly in cases with large kidney stones, and is associated with certain ergonomic challenges for urologists [5].

Maintaining a fixed position by holding the scope during fURS procedures, often by standing and manipulating various instruments, can lead to musculoskeletal problems and pain among practitioners [6]. Owing to the reported prevalence of musculoskeletal disorders among urologists performing endourological interventions, the significance of ergonomic considerations in endoscopic procedures has gained increasing attention in the medical community.

The EasyFlex system, an endoscopic surgical apparatus consisting of a doctor’s endoscopy chair and an endoscope carrying/manipulation system (International Patent No. WO2022/039688 A1), was developed to address the ergonomic limitations of traditional fURS. fURS procedures are often lengthy and commonly performed in a standing position with suboptimal posture while wearing protective fluoroscopy equipment, which may lead to surgeon fatigue, distraction, hand tremor, and decreased procedural efficiency [7, 8]. To overcome these challenges, our team designed EasyFlex system that provides a more ergonomic and controlled working environment for endourologists during endoscopic interventions.

This study focused on the validation of EasyFlex, which is designed to improve the ergonomic conditions for physicians during endoscopic procedures such as fURS. The aim of this study was to reduce the ergonomic challenges associated with traditional endoscopic procedures and to achieve improved surgical outcomes through its user-friendly design and functionality.

## Methods

The EasyFlex was developed and validated in a prospective study conducted between February 1, 2024, and November 30, 2024, at three medical centers (Sincan Training and Research Hospital, Ankara Bilkent City Hospital, and Sancaktepe Training and Research Hospital). The study evaluated the surgical and ergonomic impact of the EasyFlex system compared with conventional fURS techniques. Patients were assigned to either the EasyFlex or conventional group using a computer-generated sequence with block randomization. The allocation sequence was applied consecutively to eligible patients at each participating center. Allocation concealment was not implemented, and surgeons were aware of the assigned intervention at the time of the procedure. The procedures were performed by five experienced endourologists. Each surgeon performed procedures in both the conventional and EasyFlex groups, and surgeon-specific case distribution was recorded. Prior to the study, all participating surgeons received a demonstration-based training session on the use of the EasyFlex system. In addition, procedures were performed using a standardized fURS technique across all surgeons, including similar patient positioning, access methods, and lithotripsy approaches. The device allows manipulation similar to conventional fURS, and no significant adaptation difficulties were observed.

The two procedures were comparatively evaluated in terms of patient demographics, operative parameters, and postoperative outcomes, including complications (Clavien–Dindo grade ≥ II). Stone-free status was assessed using ultrasonography and direct urinary system graphy, with computed tomography reserved for suspected residual fragments. Intraoperative parameters, including hematuria, were recorded; hematuria was defined as bleeding that impaired endoscopic visualization. Postoperative ergonomic assessment was performed using the Ergomini scale, a validated questionnaire designed to evaluate surgeon ergonomics during endourological procedures. The scale includes multiple domains related to musculoskeletal discomfort (including neck, back, upper and lower extremities), as well as joint and headache pain, which were graded on a 5-point Likert scale (1 = no/mild discomfort to 5 = severe discomfort) [9, 10].

Patients under 18 years of age and those with a history of open ureteral or renal surgery were excluded from the study. Patients with active urinary tract infections, coagulopathies, or missing follow-up data were also excluded.

The inclusion of an endoscopy chair with the EasyFlex apparatus allows physicians to operate in a sitting posture, as opposed to the physically demanding nature of using heavy fluoroscopy shirts. Notable features of the apparatus include 4D movable armrests allowing for easy arm movements and positioning adjustments as well as adjustable back and height segments that facilitate personalized positioning relative to the patient.

### Device components

**Fig. 1:**
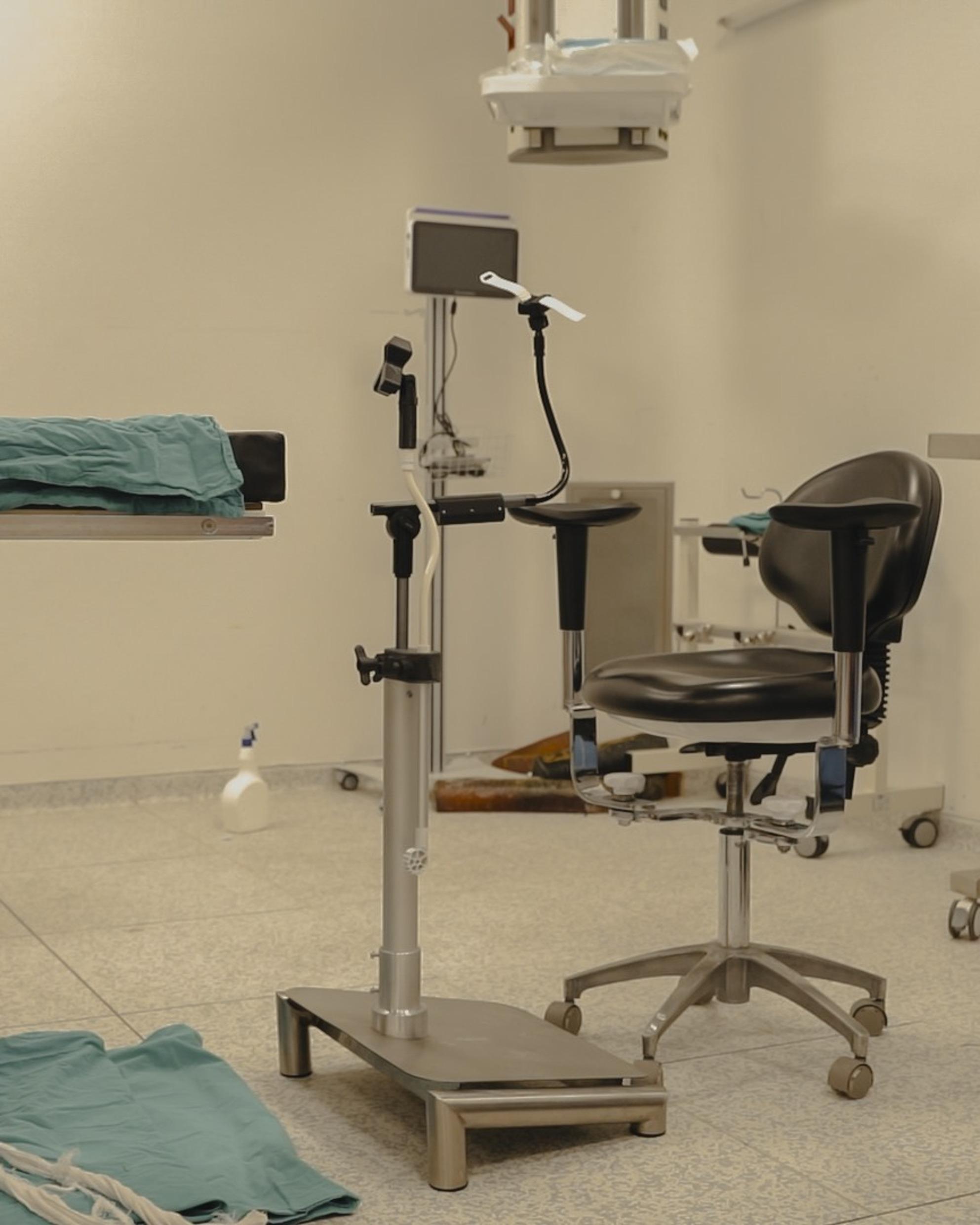
The view of the system during application in flexible ureterorenoscopy

**Fig. 2:**
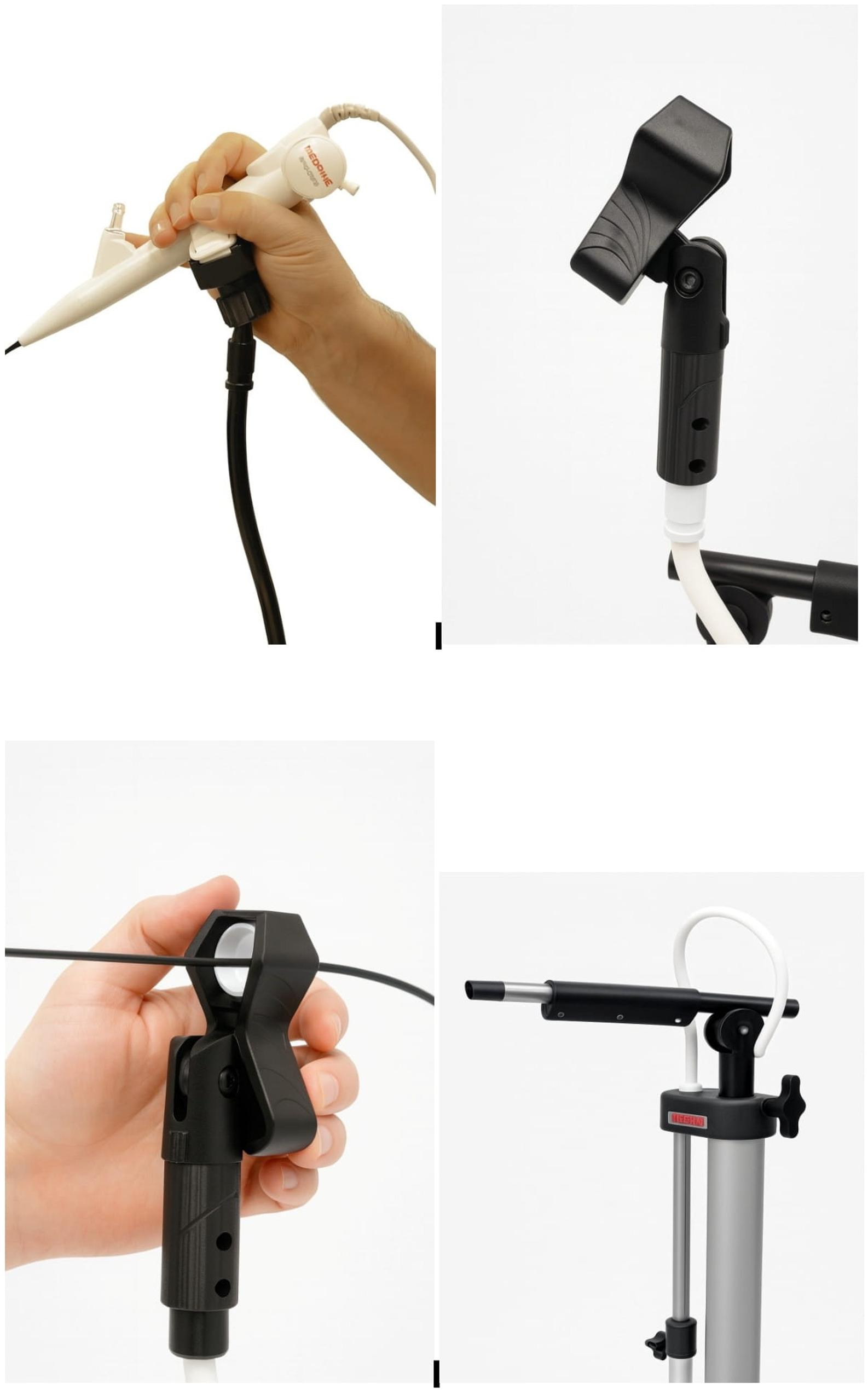
The perspective view of the system parts

The EasyFlex apparatus consists of four central elements: the endoscopy doctor chair, operating room single step, endoscope carrying/manipulation apparatus, and endoscope light tube section guiding and stabilizing apparatus (Fig. 1). The endoscopy doctor chair was designed to allow physicians to maintain an ergonomic position while conducting endoscopic procedures (video: easyflex). Meanwhile, the endoscope carrying/manipulation apparatus includes components for fixing, rotating, and manipulating the optical section of the endoscope while allowing the physician to move freely (Fig. 2a). Designed for use in the operating room, the steel single step serves as a platform for securing the endoscope carrier system, allowing physicians to position their feet in a comfortable manner. It also allows for appropriate positioning of the laser and scope pedals (Fig. 2b).

**Fig. 3:**
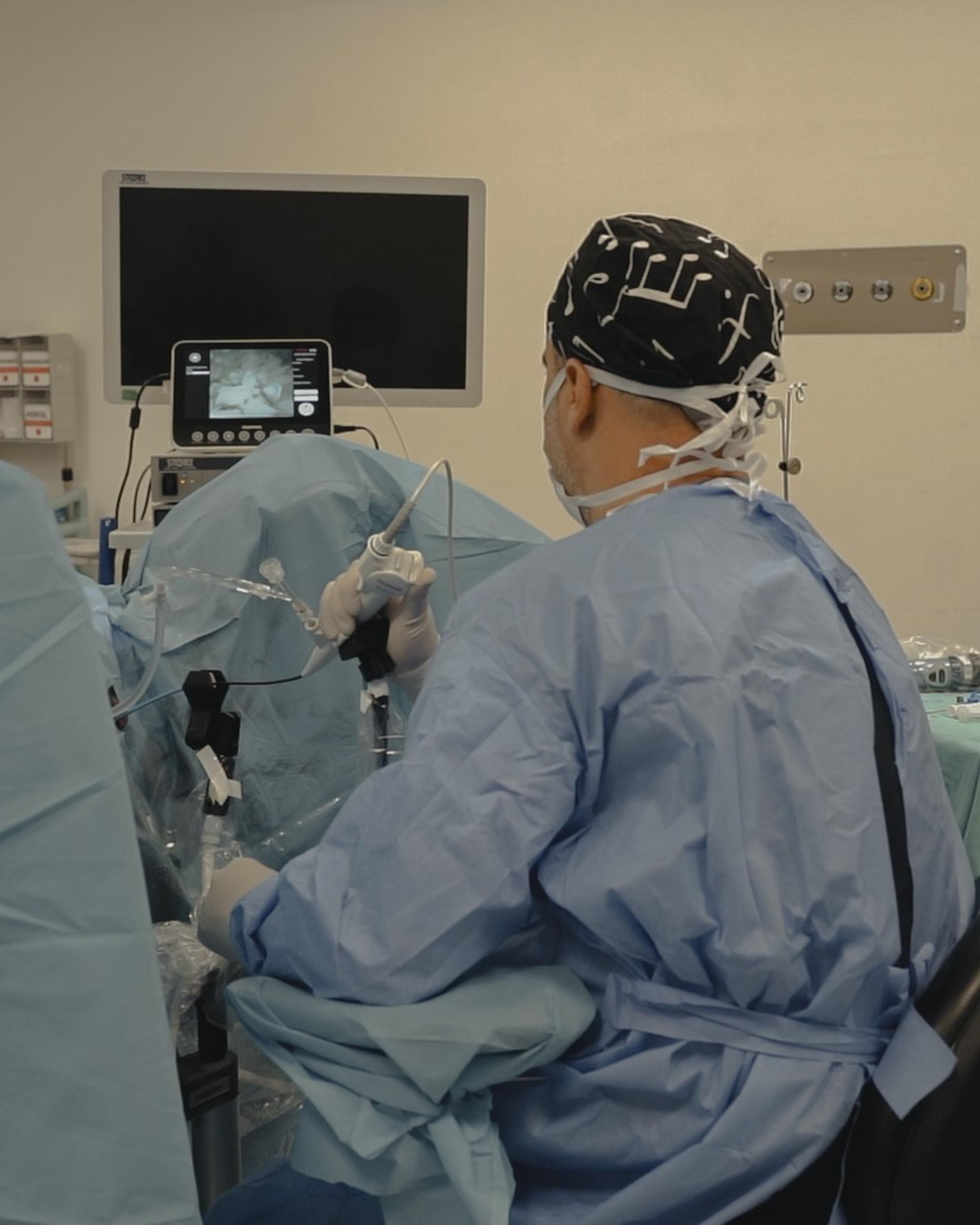
Kidney stone fragmented with the help of EasyFlex device

The light tube of the endoscope was then guided through the sheath until it reached the desired location. At this point, the endoscope optical section was firmly secured to the endoscope transport/manipulation apparatus. After positioning the endoscope carrying/manipulation apparatus and armrests to the desired configuration, the physician used the endoscope system to perform the necessary rotations and manipulations within the kidney in a highly comfortable position, as shown in (Fig. 3).

### Statistics

Data coding and statistical analyses were carried out on the computer using the SPSS 22 software (IBM SPSS Statistics, IBM Corporation, Chicago, IL). The conformity of the variables to the normal distribution was analyzed using the Shapiro-Wilk tests. Continuous variables were expressed as mean ± standard deviation (SD) for normally distributed variables and as median (minimum–maximum or interquartile range [IQR]) for non-normally distributed variables. Independent T test or Mann–Whitney U test was used to compare non-categorical parameters. Chi-square or Fisher’s exact tests were used for categorical variables.

A post-hoc power analysis was performed based on the primary outcome of ergonomic questionnaire scores. Given the large effect size observed (*r* = 0.854) and the total sample size of 168 patients, the statistical power of the study was calculated to be greater than 99% at a two-sided significance level of α = 0.05.

Effect sizes were calculated to improve the interpretability of the results. For continuous variables analyzed with the t-test, mean differences (MD) with 95% confidence intervals (CI) were reported, and Cohen’s d was calculated as a measure of effect size. Fornon-parametric comparisons (Mann–Whitney U test), effect sizes were calculated as r values using the formula r = Z/√N. For categorical variables in 2 × 2 contingency tables, odds ratios (OR) with 95% confidence intervals were calculated. For variables with more than two categories, effect sizes were assessed using Cramer’s V.

Risk factors for complications in RIRS were determined by univariate logistic regression analysis. Variables with *p* < 0.1 in univariate analysis and clinically relevant factors were included in the multivariate logistic regression model with the Backward LR method. Independent risk factors were determined as a result of multivariate analysis. A *p* value of < 0.05 was accepted as statistically significant.

## Results

In summary, 168 patients with fURS were included in the study, including 100 patients undergoing traditional treatment and 68 patients receiving EasyFlex fURS. The results revealed that the mean patient age was 46.3 yrs., with an average stone size of 14.6 mm. The postoperative stone-free rate was 73.2%, intraoperative complications were observed in 20.8% of patients, and postoperative urinary tract infections were found in 4.2% of patients.

A comparative analysis demonstrated that the EasyFlex fURS group had a higher incidence of multiple stones than the conventional group (45.6% vs. 28%, *p* = 0.019). Furthermore, the EasyFlex fURS group had a reduced intraoperative complication rate compared with the conventional fURS group (11.8% vs. 27%, *p* = 0.017). No statistically significant differences were observed between the groups in terms of operative duration, success rates, postoperative urinary tract infection rates, or length of hospital stay (*p* = 0.309 and *p* = 0.666, respectively). The results of the detailed comparative analysis are presented in Table 1.

**Table 1:** Preoperative, intraoperative and postoperative characteristics of conventional vs EasyFlex retrograde intrarenal surgery

	Total (*n*=168)	Conventional (*n*=100, 59.5%)	EasyFlex (*n*=68, 40.1%)	*p*	95% CIandeffect size
Age ( years) ( Mean±SD)	46.3±14	47±14	45.4±14	0.488^t​^	MD=1.54 (-2.829-5.903) d=0.112
Number of RIRS					
Primer, n (%)	145 (86.3)	86 (86)	59 (86.8)	0.989^f^	
Secondary, n (%)	18 (10.7)	11 (11)	7 (10.3)		Cramer’s V=0.011
Tertiary, n (%)	5 (3)	3 (3)	2 (2.9)		
lateralization					
Right, n (%)	73 (43.5)	42 (42)	31 (45.6)		
Left, n (%)	92 (54.7)	57 (57)	35 (51.5)	0.605^f^	Cramer’s V=0.085
Bilateral, n (%)	3 (1.8)	1(1)	2 (2.9)		
Stone size ( Mean±SD)	14.6±5.9	14.2±5.5	15.1±6.3	0.51^m​^	*r*=0.024
Stone number					
Single, n (%)	109 (64.9)	72 (72)	37 (54.4)	**0.019** ^**c​**^	OR=2.155 (1.136-4.805)
Multiple, n (%)	59 (35.1)	28 (28)	31 (45.6)		
Stone localization					
Pelvis, n (%)	71 (42.3)	41 (41)	30 (44.1)		
Uppercalyx, n (%)	10 (6)	5 (5)	5 (7.4)		
Middlecalyx, n (%)	18 (10.7)	12 (12)	6 (8.8)	0.882^c​^	Cramer’s V=0.084
Calyx, n (%)	46 (27.3)	27 (27)	19 (27.9)		
≥ 2 calyces, n (%)	23 (13.7)	15 (15)	8 (11.8)		
Preoperativehemogram, g/ dL( Mean±SD)	14.1±1.8	13.9±1.7	14.3±1.8	0.102^m​^	*r*=0.127
Preoperative serum creatine, mg/ dL( Mean±SD)	1±0.4	1±0.3	0.9±0.4	0.297^m​^	*r*=0.081
Comorbidities					
DM, n (%)	32 (19)	23 (23)	9 (13.2)	0.114^c​^	OR=0.51 (0.219-1.188)
HT, n (%)	36 (21.4)	26 (26)	10 (14.7)	0.08^c​^	OR=0.49 (0.215-1.117)
Thyroiddisease, n (%)	8 (4.8)	5 (5)	3 (4.4)	0.861 ^f^	OR=1.138 (0.261-4.956)
Operation time	55.4±17.6	54.7±18.9	56.5±15.5	0.309^m​^	*r*=0.078
Intraoperativecomplication presence, n (%)	35 (20.8)	27 (27)	8 (11.8)	**0.017** ^**c​**^	OR=0.361 (0.152-0.857)
Hematuria, n	13	10	3		
mucosaltear n	13	11	2		
tothe stone inabilitytoreach, n	9	6	3		
Grade A, absolute stone-free,, n (%)	97	57	40	0.561 ^c​^	OR=0.857 (0.421-1.744)
Grade B, relative Stone free(</=2 mm), n%	34	22	12	0.238 ^c^	
Grade C fragment relative stone free(2.1–4 mm), n%	12	7	5	0.414 ^c^	
Postoperativeurinaryinfectionpresence, n (%)	7 (4.2)	4 (4)	3 (4.4)	0.896 ^f^	OR=0.903 (0.195-4.184)
Fever, n	3	3	0		
Sepsis, n.	4	1	3		
Admissionduration( median [min- max ])	1 (1-18)	1 (1-15)	1 (1-18)	0.39 ^m​^	*r*=0.066

*SD* Standard Deviation, *ESWL* ExtracorporealShockWaveLithotripsy, *DM* DiabetesMellitus, *HT* Hypertension, *CI* ConfidenceInterval, *OR* Odd’sRatio, *MD* Meandifference,^*t*^ IndependentSample T Test, ^*m*^ Mann Whitney U Test, ^*c*^ Chi-square Test, ^*f*^ Fisher’s exact test

Bold *p* values indicate statistical significance

On the other hand, the evaluation of ergonomic sub-parameter scores in the conventional and EasyFlex fURS cases revealed statistically significant differences in total ergonomic scores. Conventional fURS had a higher total ergonomic score than EasyFlex fURS (*p* < 0.001). Additionally, the total conventional fURS ergonomic score for all surgeons was significantly higher than the total EasyFlex fURS ergonomic score (37.6 vs. 12.6, *p* < 0.001) (Table 2). All five surgeons contributed cases to both groups. Consistent reductions in ergonomic scores with EasyFlex were observed across all surgeons.

**Table 2:** Ergonomic questionnaire scores: Conventional vsEasyFlex retrograde intrarenal surgery by surgeon and total

Ergonomicsscore( median )	Eyebrow skeleton pain	Neck pain	Shoulder hardness	Arm pain	Front arm pain	Elbow hardness	hand pain	Wrist hardness	Finger numbness	Back pain	Leg pain	Total score
surgeon												
1st surgeon ( conventional ) *n*=20	4	3	3	3	3	3	4	3	4	3	3	36
1st surgeon ( EasyFlex ) *n*=9	1	2	1	1	2	0	1	1	1	0	0	10
*p*	**<0.001** ^**m**^											
*r*	0.866											
2nd surgeon ( conventional ) *n*=36	4	3	4	3	4	3	4	4	3	3	3	38
2nd surgeon ( EasyFlex ) *n*=24	2	2	1	1	1	1	2	1	1	0	0	12
*p*	**<0.001** ^**m**^											
*r*	0.854											
3rd surgeon ( conventional ) *n*=18	4	4	4	3	3	3	4	4	4	3	3	39
3rd surgeon ( EasyFlex ) *n*=11	2	2	0	0	1	0	1	1	1	1	0	9
*p*	**<0.001** ^**m**^											
*r*	0.855											
4. surgeon ( conventional ) *n*=13	2	2	3	3	3	3	3	3	3	3	3	31
4th surgeon ( EasyFlex) *n*=13	1	1	1	2	1	1	2	1	0	1	1	12
*p*	**<0.001** ^**m**^											
*r*	0.936											
5. surgeon ( conventional ) *n*=13	3	3	3	3	3	3	3	4	3	3	3	34
5th surgeon ( EasyFlex ) *n*=11	1	2	1	1	2	1	2	2	2	1	1	16
*p*	**<0.001** ^**m**^											
*r*	0.863											
Average ( conventional )	3.4	3	3.4	3	3.2	3	3.6	3.6	3.4	3	3	35.6
Average ( EasyFlex )	1.4	1.8	0.8	0.8	1.4	0.6	1.4	1.2	1.2	0.6	0.4	11.6
*p*	**<0.001** ^**m**^											
*r*	0.854											

^***m***^ MannWhitney U Test

Bold *p* values indicate statistical significance

Additionally, operative time was analyzed based on stone location within the kidney (pelvis, upper calyx, middle calyx, lower calyx, and complex stones). No statistically significant differences were found between the groups for any of these locations (*p* = 0.334, *p* = 0.916, *p* = 0.735, *p* = 0.571, and *p* = 0.495, respectively; Table 3).

**Table 3:** Operation time comparison by stone location: Conventional vs. EasyFlex retrograde intrarenal surgery

Case completion duration, min (IQR)				
	Conventional	EasyFlex	*p* ^m​^	*r*
Pelvis	45 (45-62)	50 (45-60)	0.334	0.115
Upper calyx	45 (35-85)	57 (40-70)	0.916	0.03
Middle calyx	45 (37.5-58.7)	45 (42.5-67.5)	0.735	0.08
Lower calyx	50 (45-75)	60 (45-65)	0.571	0.08
Complex	60 (45-80)	60 (46.2-68.7)	0.495	0.14

*IQR* Interquartile Range, ^*m*^ Mann Whitney U Test

Bold *p* values indicate statistical significance

Univariate and multivariate logistic regression analysis was used to identify risk factors for complications. According to univariate logistic regression analysis, larger stone size, multiple stone number, longer operation time and the use of conventional fURS were risk factors associated with complications. Variables with *p* < 0.1 in univariate analysis and clinically relevant factors were included in the multivariate logistic regression analysis. Accordingly, larger stone size (OR = 1.073; 95% Cl = 1.002–1.149; *p* = 0.043), multiple stone number (OR = 2.448; 95% Cl = 1.038–5.77; *p* = 0.041) and the use of conventional fURS (OR = 3.725; 95% CI = 1.455–9.535; *p* = 0.006) were found as independent risk factors for infective complications (Table 4).

**Table 4:** Determination of risk factors associated with complications in retrograde intrarenal surgery by logistic regression analysis

Univariate			Multivariate		
	OR (95% CI)	*p*	OR (95% CI)		*p*
Age (per year)	0.992 (0.966-1.02)	0.583			
Number of RIRS (non-primary)	1.412 (0.511-3.899)	0.506			
Stone size (per mm)	1.101 (1.036-1.171)	**0.002**	1.073 (1.002-1.149)		**0.043**
Stone number (multiple)	2.376 (1.113-5.071)	**0.025**	2.448 (1.038-5.77)		**0.041**
Stone location (ref. pelvis)					
Upper calyx	1.115 (0.212-5.879)	0.898			
Middle calyx	0.558 (0.114-2.73)	0.471			
Calyx	1.758 (0.729-4.235)	0.209			
≥ 2 calyces	1.239 (0.389-3.95)	0.717			
Preoperative hemogram (per g/ dL)	0.872 (0.706-1.079)	0.207			
Preoperative serum creatine (per mg/ dL)	1.136 (0.428-3.014)	0.798			
Precence of DM	1.08 (0.424-2.752)	0.872			
Precence of HT	1.646 (0.704-3.849)	0.25			
Precence of thyroid disease	1.283 (0.247-6.65)	0.767			
Operation time (per min)	1.027 (1.006-1.049)	**0.013**	1.021 (0.998-1.045)		0.072
Device (EasyFlex)	2.774 (1.174-6.553)	**0.02**	3.725 (1.455-9.535)		**0.006**

*CI* Confidence Interval, *RIRS* Retrograde Intrarenal Surgery, *DM* Diabetes Mellitus, *HT* Hypertension. Bold *p* values indicate statistical significance

## Discussion

The EasyFlex apparatus was developed and manufactured to improve ergonomics and surgical comfort during long-lasting endoscopic procedures during stone surgery. A well-planned and validated study by expert urologists across three clinics and five distinct endoscopic surgeries effectively established the significance and success of the surgical procedure.

Literature data revealed that the prevalence of orthopedic complaints among endourologists was 64.2%, which included back problems (38.1%), neck problems (27.6%), hand problems (17.2%), and hip and knee problems (14.2%). This risk is significantly higher among older endourologists (> 40 years), those of African ancestry, individuals with a longer practice tenure (> 10 years), and those conducting a higher number of annual procedures [8]. The ergonomic principles of endourological procedures as well as the overall lack of ergonomic knowledge among endourologists remain to be elucidated. The outcomes of some recently published studies have shown the importance of ergonomics during fURS procedures and musculoskeletal ergonomic scores tailored for laparoscopy–endoscopy surgeries have been used for evaluation [8–10]. In the present study, five independent endourologists performed procedures using both conventional fURS and the EasyFlex system, demonstrating a considerable improvement in ergonomic scores in favor of the EasyFlex system. The EasyFlex device has been shown to reduce foot- and eye-related symptoms by addressing ergonomic challenges. Although mild hand–arm–shoulder symptoms persist, they have been found to be significantly less severe than those associated with traditional surgical procedures. Unfortunately, studies comparing robotic fURS with traditional methods have found that the traditional approach produces higher ergonomic symptom scores [11–13].

Performing these procedures in a consistent and intensive manner in the field of endourological surgery with concern for chronic musculoskeletal symptoms poses not only a significant challenge for urological surgeons but also increases the risk of developing chronic health issues in the future [14]. The EasyFlex validation study aimed to raise ergonomic awareness and propose solutions for these challenges.

The EasyFlex system allows physicians to perform endoscopic procedures with reduced arm strain by including two distinct rotating mechanisms to improve endoscope manipulation capabilities. Physicians can delegate control of the optical section of the endoscope to the carrier system, which allows increased dexterity while performing additional manipulations. This feature maintains the endoscope’s last position, thereby potentially improving procedural control. Additionally, as another advantage the light tube and sheath guiding apparatus of the system increase physicians’ flexibility and reduce shoulder pain and stiffness associated with traditional approaches [15, 16].

If the endoscope sheath is secured during the procedure, physicians may not face sheath displacement issues. A height-adjustable operating room step makes it easier to operate auxiliary units such as laser and/or fluoroscopy device pedals. It has been well stated that surgeon’s ergonomics, surgical team composition, appropriate instrumentation, and operating room layout are important factors to consider for successful fURS procedures [17].

While robotic fURS systems have been developed and clinically introduced over the last decade to solve such challenges, their effective use remains limited owing to certain underlying reasons. These systems do not perform true robotic surgical procedures but rather provide software-assisted flexible URS handling and manipulation capabilities [17, 18]. In comparison, EasyFlex is adaptable and may have potential applicability in other endoscopic procedures; however, this remains speculative and requires further investigation [18, 19].

Related to this issue, while several endoscope holder systems have been introduced so far, many have failed to meet the specific requirements of physicians performing fURS surgeries extensively [4, 20]. Given the common use and preference for flexible endoscopy systems and surgeries among physicians, technological advancements in this field continue to influence the clinical utility of endourological management options [20, 21].

In our study, although no significant differences were observed in operative time or stone-free rates, the lower intraoperative complication rate in the EasyFlex group may be associated with improved ergonomic conditions. Enhanced surgeon comfort and stability may theoretically facilitate more controlled instrument handling; however, this relationship was not directly evaluated in the present study. Therefore, this association should be interpreted with caution, as causal relationships cannot be definitively established in the current study design. In addition, the absence of allocation concealment and the non-blinded study design may have contributed to potential bias in the observed outcomes. Notably, the EasyFlex group included a higher proportion of patients with multiple and relatively larger stones. Despite this potentially higher case complexity, comparable stone-free rates were achieved, which may indicate that the ergonomic advantages of the system help mitigate procedural challenges. Furthermore, no significant differences were observed in short-term postoperative outcomes, suggesting that the intraoperative complications were generally minor and did not have a measurable clinical impact. It should be noted that intraoperative complication rates in fURS may be influenced by multiple factors, including stone size, location, anatomy, and prior interventions. Therefore, the observed reduction in complications cannot be attributed solely to the device and should be interpreted as an association rather than a causal effect.

As an important parameter not only for the operating surgeon but also for the patients operated on, no statistically significant difference was noted regarding the operational duration values between traditional and EasyFlex fURS procedures for the management of kidney stones in all locations. At present, there are no patents addressing the specific requirements provided by the developed doctor’s endoscopy chair and endoscope carrying/manipulation apparatus. Although several surgical stools, chairs, and armchairs are available in the market, they are designed for general use rather than focusing on specific surgical procedures. Considering recent advancements in commonly performed fURS procedures, an increasing demand for such facilitating systems has become increasingly important [21].

### Limitations

This study has several limitations that should be considered. First, the EasyFlex apparatus is currently in the prototype stage, and the results may not fully reflect real-world performance in broader clinical use. Second, the study involved a relatively small sample size and was conducted across only three centers with five urologists, which may limit the generalizability of the findings. Third, the ergonomic assessments were based on subjective surgeon-reported scores without objective biomechanical or physiological measurements. Fourth, despite randomization, the study was not blinded, and the surgeons were aware of which apparatus was being used, introducing potential bias. Additionally, allocation concealment was not implemented, which may introduce selection bias. Although each surgeon performed procedures in both groups, the number of cases per surgeon was not evenly distributed, which may introduce residual operator-related variability. In addition, surgeon-related clustering effects were not specifically adjusted for in the statistical analyses. Moreover, the relatively limited number of complication events may have affected the stability of the multivariate regression analysis. Finally, the findings are limited to fURS procedures and may not be generalizable to other endoscopic or laparoscopic surgeries. In addition, as the EasyFlex system was developed by one of the authors, a potential risk of bias cannot be excluded despite the measures taken to minimize its impact.

## Conclusion

Flexible endoscopic procedures are being applied more commonly than ever in minimally invasive management of upper tract stones. Based on the need for comfortable surgery on this aspect, EasyFlex is a significant innovation designed to improve ergonomics and surgical techniques such as endoscopic procedures. Our current validation study performed by various experienced surgeons highlights the significant positive impact of EasyFlex on the musculoskeletal system, with significantly lower ergonomic scores. EasyFlex was found to be superior to traditional fURS in terms of surgeon comfort and ergonomic condition. Comparable success rates to traditional stone removal approaches for stones in all calyces were obtained with the use of EasyFlex, without requiring a longer surgical time. Although it is in the prototype stage, the EasyFlex apparatus shows a certain potential for widespread use during these procedures, with higher surgeon preference following its clinical introduction in the near future.

## Electronic Supplementary Material

Below is the link to the electronic supplementary material.

Supplementary Material 1

## Data Availability

The corresponding author makes a data access statement sharing when needed.

## Abbreviations

ESWL: Extracorporeal shock wave therapy
fURS: Flexible ureterorenoscopy
IQR: Interquartile range
PNL: Percutaneous nephrolithotomy
RIRS: Retrograde intrarenal surgery
SD: Standard deviation
